# Rupture of a caseous calcified amorphous tumour on mitral annular calcification: serial transoesophageal echocardiographic documentation

**DOI:** 10.1093/ehjcr/ytag460

**Published:** 2026-06-16

**Authors:** Haruka Harata, Takeshi Maruo, Tatsuhiko Komiya, Mitsuru Abe

**Affiliations:** Department of Cardiology, Kurashiki Central Hospital, 1-1-1 Miwa, Kurashiki, Okayama 710-8602, Japan; Department of Cardiology, Kurashiki Central Hospital, 1-1-1 Miwa, Kurashiki, Okayama 710-8602, Japan; Department of Cardiovascular Surgery, Kurashiki Central Hospital, 1-1-1 Miwa, Kurashiki, Okayama 710-8602, Japan; Department of Cardiology, Kurashiki Central Hospital, 1-1-1 Miwa, Kurashiki, Okayama 710-8602, Japan

## Case description

A 79-year-old woman with paroxysmal atrial fibrillation (CHA2DS2-VASc 4) on apixaban 5 mg twice daily, after two prior catheter ablations, presented with transient global amnesia. Comorbidities included hypertension and stage-3 chronic kidney disease (estimated glomerular filtration rate 47 ml/min/1.73 m^2^). Brain magnetic resonance imaging (MRI) showed multiple bilateral cerebral infarctions despite anticoagulation, suggesting cardioembolic stroke.

Transoesophageal echocardiography (TEE) showed a mobile, round left atrial mass (11.8 × 8.3 mm) on mitral annular calcification (MAC) (*Panel A*; Supplementary material online, *Video S1*). The mass had an iso-echoic core and a thin, smooth, hyper-echoic shell. Cardiac computed tomography demonstrated a similar 12-mm mass attached to MAC without contrast enhancement or internal calcium (86 Hounsfield units) (*Panel B, C*). Differential diagnoses included thrombus, myxoma, fibroelastoma, and vegetation; none could be definitively excluded by imaging, and definitive diagnosis would require surgical pathology.

Repeat MRI on hospital day 10 showed progressive infarctions despite anticoagulation. Surgical excision was performed on day 30. Intraoperative TEE revealed marked transformation of the mass into a linear or flat hyper-echoic structure (14 × 4 mm) (*Panel D, E*; Supplementary material online, *Videos S2*, *S3*). A grey-white calcified mass was resected (*Panel F*). Histopathology showed dense calcification with fibrin, red blood cell clusters and surface platelet thrombus, consistent with calcified amorphous tumour (CAT) (*Panel G*). Amnesia resolved postoperatively, with no recurrent embolism at 1-year follow-up.

CAT is a calcified intracardiac lesion.^1^ To our knowledge, no prior report describes a CAT with morphology overlapping caseous calcification of the mitral annulus (CCMA). We term this morphology ‘caseous CAT’. Because CCMA carries a higher cerebral embolic risk than fully calcified MAC,^2^ we hypothesize that caseous CAT shares this elevated risk. Our serial TEE captured the first imaging record of caseous CAT rupture; released material likely contributed to the cerebral emboli despite anticoagulation. Once recognized, caseous CAT warrants early surgical consideration.^3^

**Figure:**
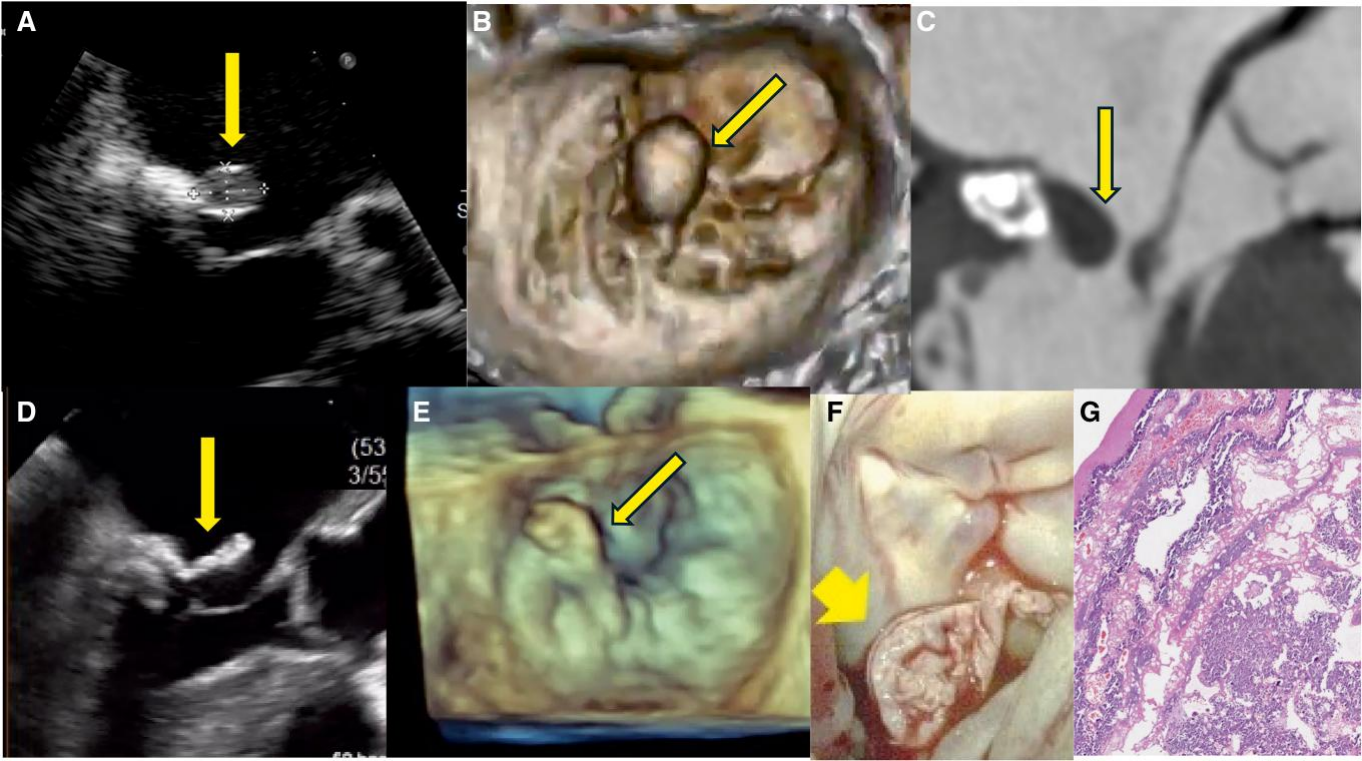
Figure. (A) Preoperative transoesophageal echocardiography (TEE) showing a mobile, round mass (11.8 × 8.3 mm; arrow) attached to mitral annular calcification, with an iso-echoic core and a thin, smooth, hyper-echoic shell. (B) Three-dimensional TEE rendering of the mass (arrow) on the posterior mitral annulus. (C) Cardiac computed tomography demonstrating a 12-mm mass attached to mitral annular calcification (arrow) without contrast enhancement. (D) Intraoperative two-dimensional TEE showing transformation of the mass into a linear hyper-echoic structure (14 × 4 mm; arrow). (E) Intraoperative three-dimensional TEE of the transformed flat structure (arrow). (F) Resected gray-white calcified specimen (arrow). (G) Histopathology (haematoxylin and eosin) showing dense calcification with fibrin and red blood cell clusters, consistent with a calcified amorphous tumour.

## Supplementary Material

ytag460_Supplementary_Data

## Data Availability

The data underlying this article are available in the article and in its online Supplementary material.

## Notes

**Consent:** Written informed consent for publication of this case, including images and videos, was obtained from the patient in line with COPE guidelines.
