# Supplementary material for: Rupture of a caseous calcified amorphous tumour on mitral annular calcification: serial transoesophageal echocardiographic documentation
Source: Eur Heart J Case Rep. 2026 Jun 16;10(7):ytag460. doi: 10.1093/ehjcr/ytag460 (PMC13327105; doi:10.1093/ehjcr/ytag460)
Supplement: ytag460_Supplementary_Data [file ytag460_supplementary_data.zip › Suppl captions.docx]

**Supplementary material**

Video S1. Preoperative TEE showing a mobile mass on mitral annular calcification.

Video S2. Intraoperative two-dimensional TEE showing the transformed linear hyper-echoic structure.

Video S3. Intraoperative three-dimensional TEE of the transformed flat structure.
